# Non-Bovine Species and the Risk to Effective Control of Bovine Viral Diarrhoea (BVD) in Cattle

**DOI:** 10.3390/pathogens10101263

**Published:** 2021-09-30

**Authors:** Caitlin A. Evans, Michael P. Reichel

**Affiliations:** 1 Institute of Future Farming Systems, School of Health, Medical and Applied Sciences, CQ University, Rockhampton 4701, Australia; 2 Department of Population Medicine and Diagnostic Sciences, College of Veterinary Medicine, Cornell University, Ithaca, NY 14853-6401, USA; bojana@gmail.com

**Keywords:** bovine viral diarrhoea, border disease, cross species infection, ruminant, cattle, sheep

## Abstract

Bovine viral diarrhoea virus (BVDV) is an economically important and highly prevalent virus of domestic cattle. Infections with BVDV may lead to both, reproductive and immunological effects that can result in widespread calf losses and increased susceptibility to diseases, such as mastitis and respiratory disease. While BVDV is generally considered to be host specific, it and other *Pestivirus* species, such as Border disease virus (BDV) in sheep, have been shown to be infecting species other than those from which they were originally isolated from. Recently BVDV was placed on the OIE’s list of notifiable disease and control and eradication programmes for BVDV have been developed throughout much of Europe, the United States, and the United Kingdom. While some countries, including Sweden and Ireland have successfully implemented eradication programmes, other countries such as New Zealand and Australia are still in the early stages of BVDV control. Despite effective control methods, incursions of BVDV into previously cleared herds still occur. While the cause of these incursions is often due to lapses in control methods, the ability of ruminant pestiviruses to infect species other than cattle poses the question as to whether non-bovine species could be impeding the success of BVDV eradication and control. As such, the aim of this review is to make mention of what is known about the cross-species transmission of BVDV, BDV and other pestiviruses between cattle and non-bovine ungulate species and draw conclusions as to the risk non-bovine species pose to the successful control and eradication of BVDV from cattle.

## 1. Introduction

Bovine viral diarrhoea (BVD) is an economically important and highly prevalent disease of cattle, found throughout cattle producing countries. Belonging to the genus *Pestivirus,* the BVD virus (BVDV) is closely related to Border disease virus (BDV) in sheep and Classical swine fever virus (CSFV) in pigs [1]. There are also a growing number of new and emerging pestivirus species identified from domestic and wild ruminants including: Bungowannah virus isolated from pigs in Australia [2]; atypical (HoBi-like) pestivirus identified in Europe, Asia and South America [3]; Pronghorn antelope virus [4] and a giraffe species from Kenya [5].

Historically, *Pestiviruses* were named after the species from which they were originally isolated; bovine pestivirus (BVDV) from cattle, ovine pestivirus (BDV) from sheep and classical swine fever virus (CSFV) from pigs [6]. Recently, however, a number of studies have indicated that pestiviruses, particularly BVDV and BDV, can infect a wide range of ungulate species, not just those from which they were originally isolated [7,8]. Additionally, infections with BVDV and BDV have been shown to trigger an antibody response in multiple species [9], primarily from the order *Artiodactyla*, which includes sheep, goats, cattle, deer and pigs as well as camels, giraffe and antelope.

Infections with BVDV and other *Pestivirus* species, can result in either acute or persistent infections in individuals. Acute infections in non-pregnant animals are generally clinically inapparent although they can result in reduced growth of young animals [10], immunosuppression and trigger the onset of other diseases such as mastitis and reproductive [11] and respiratory disease [12,13]. Additionally, infection of pregnant animals often results in severe reproductive losses due to the ability of BVDV and other pestiviruses to cross the placenta and establish an infection within the developing fetus [14,15]. When this occurs, poor conception rates, early embryonic death, abortion, stillbirth, physical malformations, and the birth of persistently infected (PI) animals are common outcomes [1,16,17].

Due to the significant costs associated with BVD outbreaks and control methods, it has recently been placed on the OIE’s list of notifiable terrestrial diseases. As a result, control and eradication programmes for BVD have been developed throughout much of Europe [17,18,19], the United States [20] and the United Kingdom [21], with other countries, such as Australia [22] and New Zealand [23] actively exploring options for control. However, there is not a “one-size-fits-all” approach to the control and eradication of BVDV [24] and the design and implementation of these control programmes must consider a number of factors, such as the prevalence of pestiviruses within the country, the density of animal populations, animal movement protocols and the accurate identification of infected animals and herds. While the identification and elimination of PI animals has been shown to be the most common and effective method for controlling BVDV [25], there have been instances where persistence of the virus has occurred, even in the absence of PI animals [26,27].

Aside from direct contact with PI cattle, there are various additional potential sources of BVDV infection in cattle, including contaminated farm equipment, flies, bedding and the boots and clothing of farm workers [28,29]. However, the ability for BVDV and other pestiviruses to cross species barriers and succeed within non-bovine hosts highlights a potential risk to eradication programmes, particularly in countries where mixed farming enterprises are common or where there are extensive numbers of wild ungulate species. As such, the aim of this review is to make mention of what is currently known about the cross-species transmission of BVDV and other pestiviruses between cattle and non-bovine ungulate species and draw conclusions as to what risks non-bovine species and non-bovine pestiviruses pose to the successful control and eradication of BVDV in cattle.

## 2. Bovine Viral Diarrhoea Virus in Non-Bovine Species

Since its first description in cattle in 1946 [30,31], infections with BVDV have been identified in multiple non-bovine ungulate species including sheep [32], goats [33], deer [9], camels [34], alpaca [35], pigs [36] and a wide array of ungulate wildlife [9]. These reports of BVDV infection in non-bovine ungulates raises concerns around the extent to which BVDV impacts the productivity of these species, the role farmed, feral and wild ungulate species may play in the spread and transmission of BVDV and the effect cross species transmission of BVDV has in the development of control and eradication programmes.

### 2.1. Sheep

Infections with BVDV in sheep have been reported since the mid to late 1970s [37] and can develop following natural transmission of the virus between cattle and sheep [38] or through experimental infection trials [39,40]. For sheep, the clinical effects following BVDV infections have been reported as being similar to those seen in BVDV affected cattle or BDV affected sheep [32]. Table 1 lists some of the clinical outcomes reported in BVDV infections in sheep, following both experimental and natural infection.

Regardless of the route of transmission, acute infections with BVDV in sheep have been shown to result in the development of BVDV-specific antibodies between 14 and 30 days after exposure [32,41]. In many cases, acute BVDV infections in non-pregnant sheep result in the absence of any observable clinical signs of infection [41]. In comparison, acute BVDV infections in pregnant ewes can lead to severe clinical outcomes. Previous studies have reported lamb/fetal losses of anywhere from 52% to 100% following infection of pregnant ewes with BVDV [32,37,41,42,43]. Studies have also reported severe neurological and physical abnormalities in lambs born to mothers infected during mid gestation with abnormalities including; arthrogryposis, brachygnathia, hydrancephaly, porencephaly and anasarca [39,44,45]. The birth of immunotolerant and persistently BVDV infected lambs has also been reported following maternal infection between 38 and 78 days of gestation [32,37,41,42,43].

Unlike in cattle, BVDV PI lambs have been reported to survive to just a few weeks of age [39,41]. Reports suggest that the risk of transmission posed by these young PI animals is low and is likely due to the presence of colostral antibodies to BVDV [46]. However, the survival of BVDV-2 PI sheep past 6 months of age has been reported [32] with the transmission of the virus to susceptible cattle and sheep being shown to occur [40]. Either way, the presence of any BVDV PI lamb on a farm poses a potential risk to the spread and persistence of the virus.

In many countries, beef cattle and sheep production overlap in terms of land use, highlighting areas where transmission may occur between neighbouring properties. Additionally, for countries such as Australia and New Zealand, the management and co-grazing of cattle and sheep on the same property is common [47], while in many European countries, alpine summer pasturing of livestock can lead to the co-mingling of ruminant species from multiple properties [48,49]. This close affinity of cattle and sheep makes it possible for interspecies transmission of BVDV (or BDV) to occur, leading to not only the persistence of the virus, but also to potential misclassifications of *pestivirus* infections [50,51], as well as increased costs associated with outbreaks [38].

Recently, a study from New Zealand reported on the seroprevalence of antibodies to pestivirus in sheep co-grazed with beef cattle. This study identified 11% of properties (2 of 18) sampled had animals testing positive for pestivirus antibodies, with 100% of animals from one property identified as serologically positive [47]. Both affected properties in this study were known or suspected of having BVDV positive cattle and highlights the potential of cross-species transmission in co-grazing situations. In addition, sheep flocks housed with or grazed with cattle have been shown to have a much higher prevalence of antibodies to BVDV than sheep managed separately from cattle [52,53]. Infections with BVDV have been identified in sheep in many countries, including Australia [54], the UK [55], Austria [56], Sweden [57], Spain [38,58], Algeria [59], Tanzania [60] and the US [61].

**Table 1:** Selected outcomes of natural or experimental infection with BVDV in sheep (MD–Mucosal Disease).

BVDV Strain	Cause of Infection	Outcome	Reference
BVDV-1 and -2	Unknown natural infection	Antibody and virus detected, lambs with low birth weight, poor growth, sporadic abortions	[62]
MD	Experimental infection via intravenous inoculation	Antibody response in ewes, poor lambing rates, PI lamb born	[37]
BVDV-2	Unknown natural infection	Abortions	[38,58]
BVDV	Experimental infection via intravenous inoculation	Antibody response in ewes, high rate of abortions and fetal deaths, low birth weights of lambs	[42]
BVDV	Experimental infection via inoculation	Arthrogryposis, brachygnathia, anasarca, porencephaly, hydrancephaly, cerebellar hypoplasia, leukoencephalomalacic lesions,	[45]
BVDV-2	Experimental infection via intravenous and intranasal inoculation	Antibody response and severe ulcerative placentitis in ewes, poor lambing rates, birth of PI lamb	[32]
BVDV-1c	Experimental infection by subcutaneous inoculation	Antibody response in ewes, poor lambing rates, birth of PI lamb	[41,43]
BVDV-1 a, b	Natural transmission from cattle	Weak lambs and mandibular brachygnathia	[55]
BVDV-1	Natural transmission from cattle	Serial generation of PI individuals (cattle–sheep–cattle–sheep)	[40]

### 2.2. Goats

In contrast to sheep, where the majority of reports of pestivirus infections are affiliated with BDV-specific antibodies, pestivirus infections in goats are more often associated with antibodies that are either not clearly specified or more closely associated with BVDV [63,64]. The first reports of BVDV infection in goats occurred as early as the 1970s [65]. Since this time, infections with BVDV have been shown to develop in goats due to both experimental and natural sources [1,33,66,67] with acute infections often resulting in seroconversion between 21 and 42 days after exposure, mild fever but otherwise no to mild clinical signs [33]. However, as with cattle and sheep, infection of pregnant does with BVDV has been shown to lead to severe reproductive losses, with up to 100% of does exposed to and infected with BVDV aborting or delivering kids that died within one to two hours after birth [1,33]. Table 2 lists some of the clinical outcomes reported in BVDV infections in goats.

Although abortion rates associated with BVDV infections in pregnant does are high, there have been reports of PI kids being born following maternal infection between 17 and 38 days gestation [33]. Overall BVDV PI kids have been reported following experimental infection [67], natural infection [33] and accidental infection via a contaminated Orf vaccine [68]. However, as with sheep persistently infected with BVDV, the long term survival of BVDV PI goats remains questionable due to the developmental and physiological issues associated with persistent infection [33].

Infections with BVDV in goats have been reported from Turkey [69], China [70,71], Peru [72], Austria [73], India [74] and Poland [75]. The prevalence of BVDV infection in goats is not well documented; however, seroprevalence rates as high as 31.3% (n = 80 flocks) and 54% (n = 63 flocks) have been reported for Austria [73] and India [74], respectively. As with sheep, it has also been reported that the seroprevalence of antibodies to BVDV is higher in goat herds that have had direct contact with cattle [63,73,74].

**Table 2:** Selected outcomes of natural or experimental infection with BVDV in goats.

BVDV Strain	Cause of Infection	Outcome	Reference
BVDV-1	Unknown natural infection	Antibody and virus detected, kids with low birth weight, poor growth, sporadic abortions	[62]
BVDV-1	Natural transmission, possibly from cattle	PI goat kid detected	[76]
BVDV	Experimental infection via intramuscular inoculation	Antibody response in does, high rate of reproductive failure	[77]
BVDV	Infected vaccine	Antibody response in does, severe reproductive failure	[68]
BVDV-1	Natural infection via experimental exposure to PI calf	Antibody response in does, high rate of abortion, two PI kids	[33]
BVDV-1	Natural infection via exposure to PI goat	Antibody response in does, development of PI kids	[33]
BVDV-1b	Natural infection via experimental exposure to PI calf	Antibody response	[66]
BVDV-2a	Natural infection via experimental exposure to PI calf	Antibody response	[66]

### 2.3. Camelids

Camels, alpacas and llamas are also susceptible to infections with BVDV and can develop BVDV-specific antibodies and BVDV related clinical signs, following exposure (Table 3). Reports of BVDV infection in alpacas and llamas include those from Australasia [78,79,80], the UK [81] and the US [35,82]. For these small camelids, acute BVDV infections are similar to those reported in cattle and sheep, often going undetected or observing only mild clinical signs such as lethargy, anorexia or a break in the fleece [83]. However, unlike some cases of BVDV infection in cattle and sheep, diarrhoea does not appear to be a feature of acute BVDV infections in alpaca [79,83].

As acute BVDV infections in alpaca typically go undetected, it is only the more chronic or persistent forms of infection that are reported. Common signs of these chronic or persistent BVDV infections in alpacas include chronic ill-thrift, poor weight gain or being underweight, severe nasal discharge and pneumonia [83,84]. Similar to cattle and sheep, acute BVDV infections can also result in reproductive losses in pregnant alpacas, such as early embryonic death, abortion [85], stillbirth, premature birth [82,83]. The births of persistently BVDV infected alpaca cria have also been reported [84,86].

The identification of BVDV in camels is limited and has mainly been reported in smaller seroprevalence surveys in some countries, including China [34], Turkey [87], Algeria [88], Iran [89] and Saudi Arabia [90]. The Algerian study is particularly noteworthy however, as viral antigen (persistent or active infection) was detected in 41.4% of camels tested. Reports on the clinical outcomes of BVDV infections in camels are inadequate but infections have been linked to both reproductive and congenital issues [91].

**Table 3:** Selected outcomes of natural or experimental infection with BVDV in camelids.

Species	Strain	Cause of Infection	Outcome	Reference
*Alpaca*	BVDV	Natural infection	Antibodies to BVDV detected	[35]
	BVDV-1b	Natural infection	Anorexia and lethargy in acutely infected animals, abortion, birth of PI cria	[83]
	BVDV-1	Natural infection	Persistent infection of cria	[84]
	BVDV-1	Natural infection	Antibodies to BVDV detected, stillbirths, congenital disease and stunted growth of cria	[92]
	BVDV	Natural infection	Antibodies to BVDV detected	[80]
	BVDV-1b	Natural infection via experimental exposure to PI alpaca	Antibodies to BVDV detected, mild clinical signs including nasal discharge and elevated body temp	[93]
	BVDV-1c	Natural infection via experimental exposure to PI heifer	Antibodies to BVDV and viral antigen detected, no clinical signs	[79]
*Llama*	BVDV-1	Experimental infection	Antibodies to BVDV detected, no signs of disease, abortion	[82]
*Camel*	BVDV	Natural infection	58.7% of camels tested in Turkey (n = 92) were positive for BVDV antibodies	[87]
	BVDV	Natural infection	Twenty seven of the 137 camels (19.7%) in Iran positive for BVDV antibodies	[89]
	BVDV	Natural infection	Abortions and uterine infection, conception failure, repeat breeding	[90]

### 2.4. Other Ungulate Species

Sheep, goats and smaller camelids (alpacas) are frequently farmed in many countries, thus allowing for increased opportunities for contact with cattle populations and potential exposure to and spread of BVDV. However, there are a large number of additional, and often wild or feral, ungulate species which are also be susceptible to BVDV infection.

It has been widely reported that multiple deer species (red deer, white-tailed etc., both wild and farmed) are susceptible to BVDV infections with many prevalence studies identifying animals positive for BVDV antibodies. While the numbers sampled have generally been small, seroprevalence studies have detected antibodies to BVDV in the range of 3% of deer from the eastern seaboard of Australia [94] to 63.5% in Mexican white tailed deer [95]. In addition, deer identified as positive for antibodies to BVDV have been detected in countries including Austria [96], New Zealand [97], Denmark [98], Spain [99], Germany [100], the UK [101] and the US [102,103,104,105]. Although the majority of the studies of BVDV infection in deer report on serological surveys, acute infections in deer have been associated with lymphopenia, pyrexia, lethargy, coughing [106] and an otherwise absence of clinical disease [107]. High rates of pregnancy loss have been associated with BVDV infections in pregnant does [108], however one study from New Zealand, while recording medium-high antibody prevalence, found no association with abortion [97]. The birth of BVDV PI fawns has also been reported [108,109], although their occurrence and subsequent survival appears to be rare.

In the US, antibodies to BVDV-1 and -2 have been detected in Rocky Mountain bighorn sheep (*Ovis canadensis, canadensis*), and sympatric mountain goats (*Oreamnos mericanum*) from adjacent mountain ranges in Nevada, at seroprevalence levels of 81% (n = 32) and 100% (n = 3), respectively [102]. Captive Rocky Mountain bighorn sheep have also been shown to contract BVDV from a contaminated modified-live Bluetongue virus vaccine [110]. This outbreak resulted in lethargy, decreased feed intake, mild dyspnea, haemorrhagic diarrhoea and death of vaccinated animals.

Infection of both American Bison (*Bison bison*) and Canadian Bison (*Bison bison bison)* with BVDV has been shown to occur with outcomes including pregnancy losses, enteritis, severe foot lesions [111], general un-thriftiness and persistently BVDV infected individuals [112]. Similarly, antibodies to BVDV have also been detected in water buffalo (*Bubalus bubalis*) from Australia [113], Italy [114], Zambia [115] and Brazil [116], but not in South East Asia (Laos and Cambodia) [117]. While the detection of BVDV-specific antibodies indicates that ater buffalo are susceptible to BVDV infections, the clinical outcomes relating to these infections are not well documented. In addition, there have also been reports of pigs infected with and developing antibodies to BVDV [36,118,119,120], which indicates the potential for confusion when diagnosing pestiviruses in pigs, due to the close association of BVDV and CSFV.

Finally, antibodies to BVDV have been reported in an array of ruminant wildlife including Ibex (*Capra pyrenaica*) from Switzerland [121]; Giraffe (*Giraffa*), Eland (*Taurotragus oryx*), Wildebeest (*Connochaetes)*, Bushbuck (*Tragelaphus scriptus*) and other ungulate species from Zambia [115]; Chamois (*Rupicapra Blainville*) from Italy [122] and Caribou (*Rangifer tarandus*) from Alaska [123].

## 3. Non-Bovine Pestiviruses in Cattle

Infections in cattle with non-bovine pestivirus isolates is not a novel occurrence. Some of the first reports of non-bovine pestivirus infections in cattle were published over 30 years ago and relate to BDV infections [124]. Since this time, naturally occurring non-bovine pestivirus infections in cattle, while not common, have been reported in countries, including New Zealand [125], the United Kingdom [126,127], Austria [128,129] and Mexico [130].

Various outcomes following non-bovine pestivirus infections in cattle are shown in Table 4. Most reports of non-bovine pestivirus infections in cattle relate to BDV infections, which can develop as a result of experimental infection or following close contact with persistently infected sheep [51,124]. Infections with BDV have led to seroconversion in infected cattle between 20 and 51 days after exposure [50,131,132], although acute infections may go undetected without serological testing. In general, the clinical signs typically identified and associated with BDV infections in cattle relate to reproductive losses. One inoculation study found that 90% of pregnant heifers infected with BDV resulted in fetal losses [124] while natural infection with BDV has resulted in a pregnancy rate of 23% [125]. Similarly, high abortion rates [133] and the birth of persistently BDV infected calves [125] have also been reported following BDV infections in cattle.

In general, outcomes of infection with BDV in cattle appear similar to those associated with BVDV and explain why non-bovine pestivirus infections often go misclassified in cattle. For example, in New Zealand in 2012, poor reproductive performance in a herd of dairy heifers was originally thought to be due to an outbreak of BVDV. It was later confirmed that a bull persistently infected with an isolate from the BDV-1 group had been purchased and used for the season and the reproductive losses observed were thus due to BDV [125]. This study highlights the ease with which misdiagnosis of pestiviruses in cattle can occur and the importance of diagnostic testing. Other instances of cattle-to-cattle transmission of BDV have been reported, including the co-mingling of a BDV PI calf with six pregnant heifers resulting in seroconversion in all heifers and persistent fetal infection in three calves [132].

In addition to BDV, Hobi-like viruses, sometimes referred to as atypical viruses or Pestivirus H, are an emerging group of pestiviruses which have recently been reported in cattle from countries including India [134] Brazil [135,136], central China [137], Italy [138,139] and Bangladesh [140]. Hobi-like viruses were first isolated from a batch of contaminated calf serum from Brazil [141] and since then it has been reported that more than 30% of fetal calf serum developed in Brazil and tested in Europe, has been contaminated with Hobi-like viruses [142]. Despite this, infections with Hobi-like viruses have also been reported following both natural [139,143] and experimental infections [144,145].

The clinical signs associated with infections with Hobi-like viruses have been shown to be similar to those typically affiliated with BVDV-1 and -2 infections (Table 4). These signs can include fever, coughing, respiratory disease, nasal discharge and acute gastroenteritis [135,139,146] but infections can also go undetected [147]. As is typical with pestivirus infections, the infection of pregnant females with Hobi-like viruses has resulted in abortion, poor pregnancy rates, the birth of persistently infected calves [135,146] and may lead to mucosal disease [138] or death in young calves [139].

**Table 4:** Selected outcomes of infection with non-bovine pestiviruses in cattle.

*Pestivirus* Species	BVDV Strain	Cause of Infection	Outcome	Reference
BDV	BDV	Experimental inoculation	Antibody response in heifers, abortion, severe growth retardation of fetuses	[124]
	BDV-1a	Mating with BDV PI bull	Antibody response and low pregnancy rate in heifers	[125]
	BDV	Mixed grazing with sheep	Antibody response in cattle	[49]
	BDV	Experimental infection by oral inoculation	Antibody response in one calf	[148]
	BDV	Co-mingling with PI sheep	Antibody response in calves	[148]
	BDV-3	Natural transmission from PI sheep	Antibody response in heifers, >50% abortion rate and the birth of a PI calf	[133]
	BDV	Natural transmission from PI calf	Antibody response in heifers, virus positive fetuses	[132]
Hobi-like		Natural infection via experimental exposure to PI heifer	Antibody response, lymphopenia	[149]
		Experimental infection	Antibody response, fever, abortion, calves with bloody diarrhoea, birth of PI calf	[144]
		Natural infection	Fever, cough, nasal discharge, elevated pulse and respiration rates, leukopenia and death in calves	[139]
	SV478/07	Experimental infection	Antibody response, fever, lymphopenia, anorexia, diarrhoea, respiratory sigs, ocular discharge	[150]
	SV757/15	Experimental infection	Antibody response, fever and lymphopenia	[150]

## 4. Threats to BVDV Control in Cattle

The ability and occurrence of cross-species transmission of pestiviruses suggests that non bovine ungulate species may present a dual threat to BVDV control or eradication programmes in cattle. Firstly, there is the potential for non-bovine species to act as residual reservoir populations for BVDV infections, allowing for BVD virus to flow back into previously cleared cattle herds. Secondly, non-bovine populations may themselves be infected with alternate pestiviruses, such as BDV, which in turn may infect cattle, creating additional sources of ill-thrift and reproductive loss in cattle.

The first major concern with the reports of BVDV infections in non-bovine species is that control or eradication efforts for BVD in cattle may be less effective if significant reservoirs of BVDV remain in other host populations. The birth of persistently BVDV infected non-bovine ungulates presents an opportunity for these residual reservoirs to reintroduce BVDV into cattle populations, at a later date.

This situation is likely to present a particular problem when the prevalence of BVDV in the respective cattle populations is nearing elimination and presents an element of concern for countries or producers relying on a positive eradication status. A significant interaction of cattle with large populations of alternative farmed ungulate species appears to have the potential to occur in a number of countries (Table 5). For example, in Australasia, both Australia and New Zealand have large populations of both sheep and cattle as well as extensive numbers of feral ungulate species. In both countries sheep and cattle are often co-managed and often co-grazed, highlighting a potential for cross-species transmission of pestiviruses in these regions [47].

In general, the prevalence of BVDV antibodies in Australian and New Zealand cattle populations is very high (~80% antibody prevalence) as BVDV is largely uncontrolled (Table 5). In comparison, the available evidence suggests that BVDV exposure of sheep is low [47,54]. It would therefore appear that infection of sheep with BVDV in these countries is a relatively rare event, one which would be even less frequent in countries with lower prevalence of BVDV and smaller populations of cattle and sheep (Table 5). A recent study, which aimed to mimic the opportunity for neighbouring transmission of BVDV from sheep to cattle, found that transmission from a PI lamb to naïve cattle failed to occur [46]. This same study highlighted potential issues surrounding the vitality of the PI lamb and does not infer that transmission from sheep to cattle would not be possible with an older, weaned PI sheep. So, although the reproductive losses associated with BVDV in sheep can be devastating, the birth and long-term survival of ovine PI animals is rare and unlikely to result in any significant further damage [41]. It is therefore likely that, in typical farming regions, where contact of cattle and sheep may be less frequent or intensive than that which is needed for effective and frequent cross-species transmission of pestiviruses, the risk sheep populations pose to the transmission and persistence of BVDV in cattle is low. However, in farming systems which co-graze cattle with sheep there may still be sufficient opportunity for spill-over of BVDV into sheep [47]. As pregnant cattle are the predominant management group which should be protected from exposure to BVDV, so as to reduce the potential for PI development, management practices ought to be implemented in these systems which reduce the exposure of pregnant cattle and sheep during pregnancy.

An alternate ungulate species, susceptible to BVDV infections and which can be found in high numbers in some countries (Table 5) is goats. In countries with large goat populations, the interaction of goats and cattle is likely to be problematic and seroprevalence rates in goats have been reported to be higher in goat herds that have had direct contact with cattle [63,73,74]. As such, for countries such as Australia, where feral goats can be found in large numbers and inhabit extensive areas of grazing land, any regular contact of feral goats with extensively managed cattle and sheep is a concern. However, as with sheep, the development and survival of persistently infected goat kids appears to be a rare event, and it is likely that the risk posed by goat populations to the maintenance and spread of BVDV back into cattle herds is low.

Due to the relatively large numbers of farmed sheep and goats in many countries (Table 5), the interaction of cattle with these species would be the most commonly expected. However, it is not just farmed ungulate species at risk of BVDV infections. There are a large number of wild and wide-ranging ungulate species which have been shown to be susceptible to infections with BVDV, with the birth of PI individuals reported in a number of these species. Often these wild non-bovine ungulates are found in exceptionally high numbers (Table 5) and for regions such as the USA, the UK and Europe, the concurrent grazing of cattle and wild ungulates across both private and public lands may provide potential for inter-species transmission of both BVDV and other non-bovine pestiviruses. It has been shown that the seasonal migration of wild ungulate species can result in their congregation during the first trimester of gestation, when PI development is possible [102]. Therefore, in countries where cattle and migratory ungulate species can gather it is advisable that only non-pregnant or vaccinated animals be allowed to graze in these situations. Finally, from the limited reports of BVDV in pig populations, both domestic and wild, it seems unlikely that pigs are a significant spill-over host of BVDV. This would be important for countries such as Australia which hosts a wild pig population estimated at 24 million individuals (Table 5).

The secondary risk posed by non-bovine ungulate species on control and eradication efforts for BVDV in cattle is the transmission of non-bovine pestiviruses, particularly BDV, to cattle. This has been reported, albeit rarely, and should not be ignored, especially at the tail end of a BVDV eradication campaign, where even single cases of BDV spilling from sheep to cattle might confuse and could prolong eradication efforts [51,125]. Hence, once a BVDV control campaign has reached the final stages of elimination of the virus and there are regions with the requisite factors (either large populations of non-bovine ungulates or intensive co-management of susceptible cattle and non-bovine ungulate species, or both), it would be prudent to investigate the exact source (and species) of any pestivirus infections. Identifying active sources of pestivirus infection in non-bovine populations will not only assist in eliminating future infection risks to susceptible herds of cattle but also assist in reducing the incidence of non-bovine pestiviruses in general and will benefit both cattle and small ruminant producers in the area.

It has been shown that communal pasturing of cattle with sheep can lead to the infection of cattle with BDV [49]. As such, non-bovine species may also be a considerable issue for countries which have relatively large populations of cattle and farmed non-bovine species, predominantly sheep, co-existing, irrespective of the control or eradication status of that country. Additionally, countries that have large numbers of free ranging and/or wild non-bovine species may also be at risk of persisting pestivirus infections. In these situations, it is encouraged that any recent or unexpected pestivirus infection be investigated for the exact source (and species) of pestivirus infection.

Finally, while the presence of BDV-PI cattle has been shown to result in poor conception rates in heifers [125] and the development of BDV positive fetuses [132], it is still debatable as to what the exact epidemiological role of BDV-PI cattle may be; are they an epidemiological dead end or could they be the start of continuous BDV infection within a herd, i.e., is further development of BDV-PI cattle possible? However, regardless of their role, the identification of these animals on farms should result in their removal and subsequent investigation into how their development and presence on farms came about. Eliminating all sources of pestivirus infection is essential to protect cleared and/or susceptible herds from the severe clinical and economic effects which come with an outbreak of pestivirus infection.

**Table 5:** Breakdown of prevalence (individuals (ind) and at a herd level) of BVDV in cattle populations, presence of non-bovine *pestiviruses* and population numbers of farmed and wild non-bovine species in selected countries.

Country	Cattle Population [151]	Prevalence of BVD in Cattle	Non-Bovine Pestiviruses Reported	Extensively Managed Non-Bovine Ungulates	Wild/Feral Non-Bovine Ungulates
*Australia*	26.4 million	86–89% (herd) [152,153]	BDV [154] Bungowannah virus [2]	Sheep (64 million) [155] Alpaca (350,000) [156] Goats (516,000) [157] Deer (44,000) [158]	Goats (4–6 million) [157] Pigs (24 million) [159] Camels (1 million) [160] Deer (2 million) [161] Buffalo (187,000) [162]
*New* *Zealand*	10.1 million	58–63% (ind) [163,164] 41–46% (herd) [165]	BDV [125]	Sheep (27.3 million) [166] Alpaca (26,000) [167] Deer (1 million) [168]	Pigs (110,000) [168] Deer (250,000) [169]
*United Kingdom*	9.89 million	England and Wales 5% (herd) [170] Scotland 73% (herd) [171]	BDV [127]	Sheep (33.8 million) [166] Alpaca (45,000) [156] Goats (104,000) [166] Deer (36,000) [172]	Pigs (low hundreds) [173] Deer (2 million) [174] Goats (3500) [173]
*United States of America*	94.3 million	69% (ind) 91% (herd) [175]	BDV [176]	Sheep (5.3 million) [177] Alpaca (350,000) [156] Goats (2.7 million) [177] Bison (184,000) [177] Deer (212,500) [177]	Pigs (6 million) [178] Deer (11 million) [179] Bison (40,000) [180]
*Brazil*	213.5 million	49–56% (ind) [181,182]	Hobi-like [135,136] CSFV [183,184]	Sheep (18.9 million) [166] Goats (10.7 million) [166] Buffalo (1.39 million) [166]	Deer (unknown) Pigs (unknown)

## 5. Conclusions

Infections with BVDV appear to be mostly confined to cattle populations, although in unique and locally important situations, spill-over into non-bovine ungulate hosts (i.e., sheep and wildlife) is possible. Spill-over of BVDV from cattle is most likely to occur when the prevalence of BVDV in cattle populations is high or where large populations of both cattle and the other farmed non-bovine ungulates co-exist, for instance in Australia and New Zealand. Other opportunities for infection of non-bovine ungulates with BVDV include instances where these species and cattle are managed in smaller numbers, but intensively and in close proximity, such as where communal seasonal pasturing occurs. While spill-over of BVDV into non-bovine ungulate populations is possible, the occurrence of this appears to be rare. It appears the virus is unlikely to persist in these populations and as such, is unlikely to spoil BVDV control and eradication efforts in cattle.

However, once control and eradication efforts are nearing their final stages and BVDV prevalence has reached a very low level in cattle populations, even rare instances of non-bovine pestiviruses infecting cattle may cause concern for cleared herds, confuse the epidemiological picture and prolong eradication efforts. Any suspected spill-over of non-bovine pestiviruses into cattle should therefore be carefully investigated. These instances are more likely to occur in countries where large populations of non-bovine ungulate species co-exist with cattle or are very intimately managed together.

To effectively manage the threats posed by non-bovine ungulate species to the control of BVDV in cattle, all sources of pestivirus infection in bovine populations need to be actively investigated. This will not only assist in eliminating future infection risks to susceptible cattle herds, but also assist in reducing the incidence of non-bovine pestiviruses in general, benefiting both cattle and small ruminant producers alike.

## Data Availability

Not applicable.
